# Do More of What Makes You Happy? The Applicability of Signature Character Strengths and Future Physicians’ Well-Being and Health Over Time

**DOI:** 10.3389/fpsyg.2021.534983

**Published:** 2021-05-31

**Authors:** Alexandra Huber, Angela Bair, Cornelia Strecker, Thomas Höge, Stefan Höfer

**Affiliations:** ^1^Department of Medical Psychology, Medical University of Innsbruck, Innsbruck, Austria; ^2^Institute of Psychology, Medical Informatics and Technology, University of Health Sciences, Hall in Tirol, Austria; ^3^Institute of Psychology, University of Innsbruck, Innsbruck, Austria

**Keywords:** signature character strengths, applicability, well-being, work engagement, burnout, health, medical education, future physician

## Abstract

Research on applying signature character strengths demonstrated positive effects on well-being, health and work behavior. Future health care professionals represent a group at risk for impaired well-being due to high study demands. This study investigates potential long-term protective effects on well-being. In total, 504 medical students participated in a longitudinal online study, with at least 96 providing complete data at all three time points (time lag: 1 year). Data on individual signature character strengths and their applicability, thriving (subjective and psychological well-being), work engagement, burnout, mental and physical health were collected. Longitudinal relations of signature character strengths’ applicability and well-being, mental and physical health were tested with cross-lagged panel analyses. Moreover, indirect longitudinal mediation effects via work engagement and emotional exhaustion were considered. Cross-lagged panel analyses demonstrated significant positive effects of thriving on signature character strengths’ applicability at later time points (*β* = 0.20 to 0.27) indicating that higher levels of well-being might be mandatory first to have access to one’s own signature character strengths in a naturalistic setting. Disentangling thriving, the effect was only significant for psychological well-being (t1-t2: *β* = 0.23; t2-t3: *β* = 0.27). Across all three time points, significant indirect effects via work engagement on the relation of the applicability of signature character strengths and well-being were identified (*r* = 0.15), whereas significant indirect effects on mental and physical health were only evident at t2 (both: *r* = 0.06) and t3 (mental health: *r* = 0.11). A longitudinal mediation analysis via work engagement revealed a significant indirect effect (*a^∗^b* = 0.13). These results call for further research as previous studies showed that the applicability of signature character strengths affected well-being, not vice versa. The ‘broaden-and-build’ theory (positive emotions broaden one’s consciousness and hereupon individuals build new enduring resources and skills) and the assumption of well-being in a “top-down” model (trait-like predisposition to interpret life experiences in positive ways coloring one’s evaluation of satisfaction in various domains accordingly) could possibly explain these novel results.

## Introduction

Being mentally healthy depends not merely on the absence of harm, sorrows or mental problems but is more about experiencing autonomy, self-control and -determination, meaning in life, and a process of continuous growing and personal development (Ryff, 1995). However, this view emerged quite recently as the field of psychology initially had a different mission after World War II: diagnosing, treating and curing mental impairments or damages to restore human functioning (Seligman and Csikszentmihalyi, 2000). In Maslow (1954) described neglected ‘healthogenic effects:’ ‘Everything seems directed toward preserving life and very little toward making it worth living’ (p. 284). Therefore, the research area of Positive Psychology attended in the late 1990s to factors that make life most worth living. However, human lives are marked by ups and downs but it is not about denying these downs within Positive Psychology (Peterson, 2006). Positive Psychology is about identifying life-affirming factors that fulfill individual potentials by fostering human functioning through three pillars: (1) positive subjective experiences (e.g., happiness / satisfaction), (2) positive individual traits (e.g., character strengths), and (3) positive institutions (e.g., families / workplaces; Seligman and Csikszentmihalyi, 2000; Peterson, 2006). According to literature, particularly applying character strengths substantially contributes to various well-being outcomes, e.g., in cross-sectional retrospective studies (Hausler et al., 2017a; Huber et al., 2019), at work (Littman-Ovadia and Steger, 2010; Harzer and Ruch, 2012, 2013), or in experimental settings (Seligman et al., 2005; Gander et al., 2013). Hence, it could be assumed that the applicability of character strengths will consequently lead to higher levels of well-being as well. However, to the authors’ knowledge there has been no examination over time in a naturalistic setting so far. Future health care professionals like medical students as an at-risk group for impaired well-being (e.g., Dyrbye et al., 2008, 2014) could benefit from potential protective effects of applicable individual character strengths. This applicability might function as a health-promoting factor being positively mediated by work engagement or negatively by the burnout dimension ‘emotional exhaustion.’ Therefore, this observational study aims to close the gap of missing empirical information on long-term effects of the applicability of character strengths on well-being and health in a naturalistic setting including three measurements (time lag: 1 year).

In general, the construct of well-being can be assigned to the first pillar of Positive Psychology (i.e. positive subjective experiences) and is mainly associated with two philosophies: hedonism and eudaimonia (Ryan and Deci, 2001). In the hedonistic tradition, well-being emerges from happiness, enjoyment and pleasure. Hedonistic joy always occurs when physical, intellectual or social needs are satisfied and accompanied by positive emotions (Waterman, 1993). The origins of the hedonistic theory date back to the ancient Greek philosopher Aristippus who claimed that happiness is the total number of hedonistic moments and the main aim in life is to experience maximal enjoyment. However, happiness cannot be reduced to the satisfaction of basic human needs as it is also based on individual values, goals and life circumstances (Diener et al., 1998). Summarizing, the hedonistic tradition defines well-being mainly by subjective happiness that is characterized by life satisfaction depending on what is important in one’s life in different domains (e.g., work, family, leisure, health, finances, self, or one’s group; Diener et al., 1999) as well as by the presence of positive and the absence of negative affect. Together, these constructs form the *subjective well-being* (SWB; Diener, 1984). In the eudaimonic tradition that also dates back to ancient Greek philosophers like Socrates or Aristotle, well-being emerges from realizing all those potentials an individual pursues to live life in a self-fulfilling way (Waterman, 1993). It can be described as personal expressiveness and enhancement of individual abilities to counter existential challenges in everyday life. This kind of well-being occurs when individuals strive for personal accomplishment or are actively and successfully involved in significant life tasks concerning aspects like autonomy, personal growth, environmental mastery, purpose in life, positive relations with others, and self-acceptance (Ryff and Keyes, 1995). This construct is usually labeled *psychological well-being* (PWB) in the literature. Although both concepts of SWB and PWB are related and interdependent, they are empirically distinct (Ring et al., 2007). A recently developed concept considering SWB and PWB is *thriving*, which is defined as ‘state of positive functioning at its fullest range-mentally, physically and socially’ (Su et al., 2014). The corresponding questionnaire, the ‘Comprehensive Inventory of Thriving’ (CIT), subsumes the full range of various established well-being theories and aspects (optimism: Scheier and Carver, 1985; PERMA-model: Seligmann, 2011; PWB: Ryff and Keyes, 1995; self-determination theory: Ryan and Deci, 2000).

According to the second pillar of Positive Psychology (i.e., positive individual traits), character strengths can substantiate and contribute to all aspects of well-being. Peterson and Seligman (2004) introduced the Values in Action (VIA) classification to describe the good character, representing 24 character strengths assigned to six virtues (courage, humanity, justice, temperance, transcendence, and wisdom) that have been theoretically considered important across many religions and cultures. Here, character strengths are conceptualized as positive, stable and moral traits which can be measured with the VIA-Inventory of Strengths (VIA-IS; Peterson and Seligman, 2004; Peterson and Park, 2009). However, the authors assume that the environment depending on the respective conditions can also shape these 24 character strengths. They claim that every person has about three to seven so-called ‘signature character strengths’ which are really characteristic of an individual. If people act accordingly to their signature character strengths and show corresponding behavior, they feel authentic (‘this is the real me’), energized and intrinsically motivated (Peterson and Seligman, 2004). Thus, signature character strengths can be understood as an inherent part of one’s personal identity (Forest et al., 2012). Character strengths become apparent when they are applied in conducive environments (Saucier et al., 2007). In general, one can distinguish between the possession of a character strength on a ‘trait-level’ in terms of quite stable individual differences (e.g., I am a creative person) and the application of a character strength (e.g., I am doing creative things) though being dependent on different environments (e.g., working context, private life, leisure time). Depending on these environments, the same character strengths can be used to different degrees or shown by different behaviors. Therefore, the ‘applicability’ of character strengths, as introduced by Harzer and Ruch (2013), covers the character strengths’ relevance (e.g., my creativity is useful, demanded, and/or important for me in this context) as well as the respective behavior (e.g., I behave creative). Their ‘Applicability of Character Strengths Rating Scales’ (ACS-RS) measure the applicability of the 24 VIA-character strengths in work and private life. Researchers argued that character strengths themselves already contribute to various aspects of well-being but particularly applying them is crucial (Gander et al., 2013; Huber et al., 2019).

People can experience a specific type of engagement during work exceeding the extent of what is described within well-being theories. Work engagement is defined as positive, fulfilling work-related motivational state of mind characterized by vigor (high levels of energy while working; persistence even among difficulties), dedication (strong involvement; experiencing meaningfulness at work), and absorption (fully concentrated and engrossed by work, whereby time passes quickly; Schaufeli et al., 2002). Inspired by Positive Psychology and considering personality- and health-promoting effects of work, particularly work engagement can further contribute to higher levels of well-being buffering the costs of health impairing job demands (Bakker and Demerouti, 2007). The negative counterpart would be burnout, which’… refers to the emotional depletion and loss of motivation that result from prolonged exposure to chronic emotional and interpersonal stressors on the job’ (Leiter et al., 2015). Therefore, burnout is defined as a syndrome comprising work-related emotional exhaustion (depleted emotional and internal resources; feelings to not have anything more to give to the job), depersonalization (attempt to distance oneself from the job; doubts about the value of work; actively starting to ignore positive aspects of the job), and reduced personal accomplishment (feelings of much less effectiveness; performance decreases; Maslach et al., 2001). As burnout symptoms arise by definition from work experiences, the construct, and particularly emotional exhaustion as a key dimension, is distinguishable from other clinical symptoms and syndromes implying reduced well-being in general (e.g., chronic fatigue syndrome, depression). Although work-related emotional exhaustion does involve feelings of fatigue, being used up, irritability, frustration, and wearing out (Gaines and Jermier, 1983), the chronic fatigue syndrome mostly occurs in combination with physical diseases or their treatment and inflammatory processes (e.g., cancer: meta-analysis by Ma et al., 2020; neurological diseases: Penner and Paul, 2017; rheumatic diseases: Overman et al., 2016). In contrast to job burnout, depression is more global and pervading every aspect of a person’s life, not only work (Maslach et al., 1996). However, the absence of burnout symptoms does not imply that a person fully experiences work engagement-both phenomena can occur simultaneously in positive or negative ways (e.g., Schaufeli et al., 2009). Work engagement is thereby mainly influenced by work-related resources (‘motivation path’) whereas emotional exhaustion is mainly affected by work-related stressors (‘strain path’) according to the ‘Job Demands-Resources Model’ (JD-R model; Bakker and Demerouti, 2007) which needs to be considered when deducing practical implications. Moreover, when the applicability of character strengths is seen as individual resource in the working context, particularly a positive relation via work engagement with well-being and health could be assumed.

Many people just think of employees when referring to work engagement or burnout dimensions. However, students can experience work engagement or burnout as well, as work can be defined as (1) an ‘activity involving mental or physical effort done in order to achieve a purpose or result,’ (2) ‘a task or tasks to be undertaken,’ and (3) ‘a thing or things done or made; the result of an action’ (Oxford Dictionaries, 2019). According to that, work consists of many targeted actions and refers, beside to typical paid labor in employment, also to a broader formulation (‘achieving an aim’) including, e.g., domestic work, working as parents, voluntary work or study work (Strecker et al., 2020). Therefore, students do ‘work’ at universities for the aims of obtaining professional skills or a university degree, being exposed to various demands, resources and stressors affecting their motivation, health, and well-being. In particular, medical students have to face high study demands. They reported more depressive symptoms and higher levels of distress concerning their health compared to the general United States population (Dyrbye et al., 2014), dis-satisfaction (Lebensohn et al., 2013), reduced quality of life (West et al., 2011), and other health restrictions. International burnout research focused on medical students and identified early origins of recurrent physician burnout (e.g., Kachel et al., 2020). Those studies, usually focusing on emotional exhaustion, revealed a prevalence of about 45% up to 70% to have at least once these symptoms during medical education (Dyrbye et al., 2008; IsHak et al., 2013). A study on German medical students also associated perceived stress with poor academic performance (Kötter et al., 2017). Nearly all of these studies recommended increasing awareness of the phenomenon of burnout in the study context and implementing appropriate interventions in the medical curriculum.

The VIA-character strengths have been frequently analyzed in relation to various well-being outcomes, but only little research has focused on medical students so far and none considered work engagement or emotional exhaustion as possible mediators in a naturalistic longitudinal design. Studies coming from the ‘Well-Med’ project (P27228-G22; see sample and procedure) contributed extensively to the current state of art. A study on medical students’ character strengths profiles reported *honesty, fairness, judgment, kindness*, and *love* as their five highest character strengths on average, with all character strengths being positively related to thriving and mostly to work engagement (Huber et al., 2020). The longitudinal analyses showed that *zest* positively influenced future thriving and work engagement and *self-regulation* was particularly relevant for future work engagement. Surprisingly, *appreciation of beauty and excellence, perspective, creativity* and *hope* had negative effects on thriving or work engagement (Huber et al., 2020). *Curiosity, gratitude, hope, love*, and *zest* being often identified as the character strengths most strongly related to life satisfaction (e.g., Park et al., 2004; Buschor et al., 2013), were also strongly correlated to SWB in previous studies examining medical students, whereas correlations with PWB were usually higher (Hausler et al., 2017b). Other results indicated that again *curiosity, gratitude, hope, love*, and *zest* were related to occupational well-being across a range of different professions (e.g., increased job satisfaction; Peterson and Park, 2006) and that applying character strengths at work is related to various positive experiences (e.g., pleasure, work engagement, meaning) as well as behavioral outcomes (e.g., Littman-Ovadia and Steger, 2010; Seligmann, 2011; Harzer and Ruch, 2013). The applicability of certain character strengths was influential for hospital physicians’ PWB, work engagement and burnout (Huber et al., 2019). In particular, medical students’ applicability of signature character strengths was negatively related to emotional exhaustion and significant indirect effects via emotional exhaustion on well-being, physical and mental health were found cross-sectionally (Hausler et al., 2017a). However, information on long-term mediation effects via emotional exhaustion is still missing as well as on effects via work engagement in general. Studies examining different populations but using somehow similar constructs showed that work engagement negatively mediated the relation between SWB and work withdrawal behavior (not investing efforts toward the accomplishment of organizational goals, maybe also relating to character strengths; Garg and Singh, 2019) as well as the relation between job resources (cf. the applicability of character strengths as individual resource) and psychological distress (Hopkins, 2012). Strecker et al. (2019) showed that the applicability of signature character strengths significantly mediated the positive relation between work characteristics (like social support from colleagues and supervisors) and work engagement; and in turn, that higher work engagement contributed to higher levels of well-being and health (Bakker and Demerouti, 2007). Finally, the effect of character strengths-based interventions (e.g., ‘Using your strengths in a new way every day’ for 1 week, or applying your signature character strengths over 6 months; Seligman et al., 2005) on increasing well-being and decreasing depression has been well documented over time (Gander et al., 2013; Proyer et al., 2014). Focusing on one’s strengths during psychotherapy has been more effective than common therapy methods or the additional use of antidepressants (Seligman et al., 2006). By conducting a 6-week, group-therapeutic intervention, the authors demonstrated reduced symptoms from moderate depression and higher levels of life satisfaction and remission rates over 1 year.

In summary, this study aims to close the gap of empirical knowledge on long-term effects of the applicability of signature character strengths on well-being, physical and mental health over time in a naturalistic setting of medical students. Previous cross-sectional studies indicated a positive relation and implicitly assumed causality to some extent in this direction. Examining this assumption longitudinally among medical students is important as future health care professionals could particularly benefit from this potential protective effect in their daily studies. Indirect effects via work engagement will be considered for the first time based on the ‘motivation path’ and indirect effects via emotional exhaustion that have shown their influence on the relation of the applicability of signature character strengths and well-being / health only cross-sectionally so far will be examined longitudinally (e.g., Hausler et al., 2017a; Lavy and Littman-Ovadia, 2017).

H1a: There is a positive effect of the applicability of signature character strengths on well-being (thriving, SWB, PWB) and health (physical and mental health) over time.

H1b: The applicability of signature character strengths affects well-being and health indirectly via (increased) work engagement and (reduced) emotional exhaustion within each time point.

## Materials and Methods

### Sample and Procedure

Data analyzed in this study were collected within the ‘Well-Med’ project (funded by the Austrian Science Fund) from 2015 to 2017 at a medical university in Austria. With institutional review board approval, first year medical students (human medicine; dentistry) were invited via email to complete an online survey (Limesurvey GmbH, 2003, version 2.05+). The purpose of the study was explained within this invitation including the link to the online survey. The invitation link was sent out by the university, guaranteeing data transfer by an encrypted server. Anonymity and longitudinal re-identification of the participating students was granted using a token system. Incentives comprised direct automated feedback on their individual signature character strengths, medical education credits, and a raffle of medical books and vouchers. All relevant constructs used within the analyses in this study were inquired at all three time points, always in the middle of the academic year. In total, 837 answers from 504 medical students were collected (t1: 431; t2: 267; t3: 139). Matched data across all three time points (time lag: 1 year) were available for 101 participants but due to non-complete data sets and statistical outliers, 97 (t1|t2) and 96 (t3) remained, respectively.

No significant differences in terms of sex, age, nationality, well-being, mental health, applicability of signature character strengths, work engagement, and emotional exhaustion were found when comparing the longitudinal respondents with the non-respondents. Medical students participating only at t1 reported lower physical health compared to students taking part at all three time points (Cohen’s *d* = 0.14; *p* = 0.047). In the longitudinal design, demographics were available for 97 students with 64% being female (*N* = 62) and 36% being male (*N* = 35). Their mean age at baseline was 20.3 ± 2.0 years (ranging from 17 to 28 years). More than half of them were Austrians (50.5%), followed by Germans (26.8%), Italians (21.6%), and other nationalities (1.0%). At baseline, three-fourths (74.2%) of the medical students were not in a relationship and about half of them (45.4%) lived in a shared apartment. On average, they put 37.19 ± 15.8 h (ranging from six to 70 h) per week into their medical study.

### Measures

#### Thriving (CIT)

The German version of the ‘Comprehensive Inventory of Thriving’ (Hausler et al., 2017) was used to measure well-being. It comprises 54 items rated on a five-point scale ranging from ‘strongly disagree’ (=1) to ‘strongly agree’ (=5). The items request 18 aspects of well-being, three assigned to SWB and fifteen to PWB as composite scores (see Hausler et al., 2017). SWB comprises life satisfaction, positive and negative emotions; PWB includes autonomy, engagement, meaning, mastery (accomplishment, learning, self-efficacy, self-worth, and skills), optimism and relationships (belonging, community, loneliness, respect, support, trust). Each aspect is measured with three items. An overall well-being mean value can be calculated as well, with higher scores indicating higher levels of well-being. Hereafter, when referring to this overall well-being score, the term *thriving* will be used. Cronbach’s alpha in this sample ranged from *α* = 0.92 to 0.95 across time (SWB: *α* = 0.91-0.93; PWB: *α* = 0.90-0.94). Item examples are: ‘My life is going well’ (SWB; life satisfaction); ‘There are people I can depend on to help me’ (PWB; relationship).

#### Character Strengths and Signature Character Strengths (VIA-120)

For examining character strengths, the validated German 120-item version of the VIA-IS (VIA-120) was used (Höfer et al., 2019). Medical students received automated individual feedback on their five highest character strengths based on the respective mean and were then asked to rate the ten criteria defining a signature character strength (Peterson and Seligman, 2004). Character strengths meeting these criteria (=signature character strengths) were then rated regarding their applicability. Cronbach’s alpha in this sample for the VIA-120 ranged from α = 0.59 to 0.93 (t1: *humility* and *self-regulation α* = 0.60; t2: *humility α* = 0.59; t3: *teamwork α* = 0.60; t1-t3: *spirituality α* = 0.92-0.93). The response format was a five-point scale from ‘strongly disagree’ (=1) to ‘strongly agree’ (=5), with higher scores indicating a more distinct character strength. Item examples are: ‘I always keep my promises’ (*honesty*); ‘I am never too busy to help a friend’ (*kindness*); ‘I am always willing to take risks to establish a relationship’ (*love*).

#### Applicability of Signature Character Strengths (ACS-RS)

These scales were applied to evaluate the applicability of the individual five signature character strengths at work (=study) and in private life (Harzer and Ruch, 2013). For each signature character strength, eight items (four questions referring to work and personal life each) were rated on a five-point scale from ‘never’ (=1) to ‘(almost) always’ (=5). The ACS-RS consider individual perceptions of four influences: two external (normative demands of a situation; appropriateness of certain behavior within a given situation) and two internal (perceived presence of factors that may facilitate or restrain strength-related behavior; intrinsic motivation to show certain behavior; Harzer and Ruch, 2013). Therefore, the items ask if the character strength is ‘demanded,’ ‘helpful,’ ‘important for me,’ and ‘used’ in work or private life. Within this study, only work (= study) related applicability will be reported. The internal consistency in this sample ranged from α = 0.67 (t3) to 0.84 (t2).

#### Physical and Mental Health (SF-12)

In this study, the German Short Form Health Survey including 12 items was used to assess physical and mental health of the past 4 weeks (Bullinger and Kirchberger, 1998). Eight dimensions of subjective health perception form two distinct higher-ordered clusters-the Physical Component Summary (PCS) and Mental Component Summary (MCS) measure, presented as T-scores (mean 50 ± 10) with higher scores indicating better health. Reference data from various samples are available as well as internal consistency ranging from α = 0.57 to 0.94 (Bullinger and Kirchberger, 1998). The formula to transform raw data into standardized sum scores always includes different weighted regression coefficients thus preventing calculating the internal consistency for this sample. Item examples are: ‘How much did pain interfere with your normal work including both work outside the home (studies) and housework’ (PCS); ‘How much of the time did you feel discouraged or depressed’ (MCS).

#### Work Engagement (UWES-S)

Work engagement was measured unidimensionally with the German nine-item student short-version of the ‘Utrecht Work Engagement Scale’ (Schaufeli and Bakker, 2003; Schaufeli et al., 2006) which is defined as a positive, fulfilling work-related state of mind. The response format was a seven-point scale ranging from ‘never’ (=0) to ‘always’ (=6), with higher scores indicating more engagement. Cronbach‘s α in the present study ranged from α = 0.90 (t1) to 0.93 (t3). An item example is: ‘When working for my studies, I feel strong and vigorous.’

#### Emotional Exhaustion (MBI-SS-GV)

The burnout dimension ‘emotional exhaustion’ was assessed with the adapted and modified German student version of the ‘Maslach-Burnout-Inventory’ (Gumz et al., 2013). The dimension consists of five items that can be answered on a seven-point scale from ‘never’ (=0) to ‘daily’ (=6), with higher scores indicating higher levels of emotional exhaustion. Cronbach’s alpha for this dimension ranged from α = 0.82 (t3) to 0.89 (t2) in this sample. An item example is: ‘I feel emotionally drained from my studies.’

### Statistical Analyses

For all statistical analyses, IBM SPSS Statistics 21 with AMOS Graphics (IBM Corp, 2012) and the SPSS macro PROCESS (Hayes, 2018) were used. By transforming all scores into *z*-values, potential statistical outliers (± 3.29) were identified (*N* = 3). Pearson’s coefficient inter-correlations were computed to assess the relations of the study variables, which can be interpreted as follows: *r* < 0.10 = no correlation, *r* = 0.10-0.29 = low correlation, *r* = 0.30-0.49 = moderate correlation, *r* ≥ 0.50 = high correlation (Cohen, 1988). Cronbach’s alpha indicates acceptable internal consistency when values are >0.70 (see Peterson, 1994). All longitudinal designs (multiple linear regressions) examining the long-term relations of the applicability of signature character strengths and well-being (thriving, SWB, PWB), physical and mental health were investigated by using cross-lagged-panels. The longitudinal indirect effects via work engagement and emotional exhaustion were investigated by Hayes’ macro PROCESS (2018), conducting various mediation analyses (t1-t3: confidential interval of 95%; 10.000 bootstrap samples). The macro calculates direct, indirect and total effects, with indirect effects being supposed to be significant when number ‘zero’ is not included within the 95% confidence interval (CI). Analyses of variance (ANOVAs) with repeated measures were applied to compare all means in terms of significant changes over time. To avoid any bias based on different questionnaire evaluations (e.g., T-scores vs. non-standardized means) calculations were conducted with *z*-values, respectively.

## Results

Descriptive statistics including means, standard deviations, minimum / maximum scores, skew, kurtosis, and Cronbach’s alpha are presented in Table 1, ranging from α = 0.67 (ACS-RS t3; with regard to signature character strengths overall on average at t3) to 0.95 (thriving t2|t3). The lowest internal consistencies with regard to individual signature character strengths were found for *humility* (t1: α = 0.60; t2: α = 0.59), *self-regulation* (t1: α = 0.60), and *teamwork* (t3: *α* = 0.60) indicating low reliability of the respective character strengths at that time point. ANOVAs did not reveal any significant changes between the three measurements. According to the ACS-RS, medical students perceived the highest applicability in their study life for *fairness, hope, kindness, perseverance*, and *zest* in total. Overall and at every time point, the most frequent individual signature character strengths comprised *honesty, judgment, kindness*, and *love*. At least 68.1% of the participating students reported three out of five signature character strengths to be constant over all three time points. Thereof, 20.6% reported four and 2.1% all signature character strengths to be consistent over time. There was no complete change of all signature character strengths in any participant from t1 to t3.

**TABLE 1:** Descriptive statistics over all three time points.

	***N***	***M***	***SD***	***minimum***	***maximum***	***skew***	***kurtosis***	***α***
**ACS-RS (signature character strengths)**								
t1	97	3.81	0.46	2.35	4.90	−0.41	0.87	0.71−0.76
t2	97	3.81	0.48	2.55	4.90	−0.21	−0.19	0.74−0.84
t3	97	3.75	0.46	2.80	4.85	0.34	−0.30	0.67−0.81
**Thriving**								
t1	97	4.03	0.38	2.65	4.83	−0.38	0.62	0.92
t2	97	3.99	0.43	2.96	4.87	−0.06	−0.23	0.95
t3	96	3.97	0.45	2.83	4.89	−0.09	−0.42	0.95
**SWB**								
t1	97	4.09	0.59	2.00	5.00	−0.53	0.80	0.91
t2	97	4.01	0.65	2.11	5.00	−0.64	0.57	0.93
t3	96	4.04	0.62	2.33	5.00	−0.44	−0.19	0.92
**PWB**								
t1	97	4.02	0.37	2.78	4.80	−0.29	0.35	0.90
t2	97	3.98	0.41	3.00	4.89	0.01	−0.22	0.93
t3	96	3.95	0.45	2.69	4.87	−0.15	−0.24	0.94
**MCS**								
t1	97	42.93	10.05	21.58	57.88	−0.46	−0.86	NA
t2	97	45.33	9.51	20.52	58.80	−0.66	−0.61	NA
t3	97	44.70	10.14	15.59	58.80	−0.77	−0.42	NA
**PCS**								
t1	97	56.66	5.25	37.67	66.78	−1.34	2.63	NA
t2	97	55.38	5.88	37.50	64.86	−1.13	1.41	NA
t3	97	55.75	5.92	35.16	65.93	−1.30	1.79	NA
**WE**								
t1	97	4.45	0.86	1.78	6.00	−0.67	0.37	0.90
t2	97	4.42	0.93	0.78	6.00	−1.00	1.52	0.92
t3	96	4.30	1.03	0.78	5.89	−0.68	0.12	0.93
**EE**								
t1	97	2.63	1.01	0.20	5.60	0.59	0.52	0.85
t2	97	2.69	1.13	0.20	5.60	0.21	−0.40	0.89
t3	96	2.77	0.99	0.40	5.00	0.05	−0.29	0.82

*N = number of participants, M = mean, SD = standard deviation, α = Cronbach’s Alpha, ACS-RS = applicability of character strengths-rating scales, SWB = subjective well-being, PWB = psychological well-being, PCS = physical component summary, MCS = mental component summary, WE = work engagement, EE = emotional exhaustion.*

Pearson’s coefficient inter-correlations between all relevant study variables at the three time points ranged from *r* = −0.62 to 0.99 (Table 2). Strong correlations (*r* ≥ 0.50) within the time points were found for thriving with SWB and PWB (t1|t2|t3), the applicability of signature character strengths with thriving (t3) and PWB (t2|t3), work engagement with thriving (t1| t2) and PWB (t1-t3), and mental health with SWB (t3) and emotional exhaustion (t1-t3). Only 14 out of 21 inter-correlations with physical health within one time point had a correlation coefficient > 0.10 at most up to *r* = -0.22, therefore sharing only a maximum of about 5% variance (Table 2). Post hoc G^∗^power analyses for this sample revealed a power of at least 92.7% to detect medium effects in the cross-lagged-panel designs (alpha-error: 0.05, max. sample size: 96, number of predictors: 2; Faul et al., 2009). Only for two non-significant results (out of 20 in total), the power dropped to 85.8% and 62.1%, respectively.

**TABLE 2:** Pearson’s coefficient inter-correlations between all relevant study variables.

	**ACS t1**	**ACS t2**	**CIT t1**	**CIT t2**	**SWB t1**	**SWB t2**	**PWB t1**	**PWB t2**	**PCS t1**	**PCS t2**	**MCS t1**	**MCS t2**	**WE t1**	**WE t2**	**EE t1**	**EE t2**
ACS t1	—		ACS t3-CIT t3 0.52**		ACS t3-SWB t3 0.37**		ACS t3-PWB t3 0.53**		ACS t3-PCS t3 0.08		ACS t3-MCS t3 0.17		ACS t3-WE t3 0.47**		ACS t3-EE t3 -0.17	
ACS t2	0.42**	—														
ACS t3	0.55**	0.39**	0.37**	0.39**	0.19	0.29**	0.39**	0.40**	0.23*	−0.03	0.08	0.07	0.31**	0.39**	−0.25*	−0.08
CIT t1	0.44**	0.35**	—		CIT t3-SWB t3 0.80**		CIT t3-PWB t3 0.99**		CIT t3-PCS t3 0.09		CIT t3-MCS t3 0.41**		CIT t3-WE t3 0.50**		CIT t3-EE t3 −0.35**	
CIT t2	0.25**	0.49**	0.68**	—												
CIT t3	0.35**	0.34**	0.63**	0.76**	0.46**	0.62**	0.62**	0.74**	0.06	0.19	0.37**	0.25*	0.43**	0.49**	−0.46**	−0.37**
SWB t1	0.28**	0.18	0.78**	0.50**	—		SWB t3-PWB t3 0.69**		SWB t3-PCS t3 0.06		SWB t3-MCS t3 0.58**		SWB t3-WE t3 0.35**		SWB t3-EE t3 −0.45**	
SWB t2	0.07	0.32**	0.48**	0.82**	0.50**	—										
SWB t3	0.17	0.18	0.40**	0.56**	0.37**	0.62**	0.37**	0.51**	0.06	0.27**	0.42**	0.36**	0.28**	0.35**	−0.38**	−0.39**
PWB t1	0.46**	0.37**	0.98**	0.67**	0.64**	0.43**	—		PWB t3-PCS t3 0.09		PWB t3-MCS t3 0.35**		PWB t3-WE t3 0.50**		PWB t3-EE t3 −0.30**	
PWB t2	0.29**	0.50**	0.69**	0.98**	0.47**	0.71**	0.70**	—								
PWB t3	0.38**	0.37**	0.65**	0.76**	0.45**	0.58**	0.65**	0.76**	0.06	0.15	0.33**	0.20*	0.45**	0.50**	−0.45**	−0.34**
PCS t1	0.06	−0.03	0.11	0.13	0.07	0.17	0.11	0.11	—		PCS t3-MCS t3 −0.16		PCS t3-WE t3 0.13		PCS t3-EE t3 −0.11	
PCS t2	0.10	0.05	0.26**	0.22*	0.24*	0.21*	0.25*	0.20*	0.25**	—						
PCS t3	0.05	−0.09	0.14	0.053	0.18	0.052	0.12	0.05	0.33**	0.33**	0.15	−0.04	0.10	0.18	−0.20	−0.08
MCS t1	0.10	0.10	0.37**	0.25*	0.49**	0.30**	0.30**	0.21*	−0.10	0.37	—		MCS t3-WE t3 0.28**		MCS t3-EE t3 −0.58**	
MCS t2	−0.12	0.14	0.06	0.32**	0.18	0.50**	0.02	0.24*	0.04	−0.15	0.41**	—				
MCS t3	0.07	0.08	0.21*	0.23*	0.29*	0.35**	0.17	0.17	0.03	0.28**	0.56**	0.50**	0.19	0.19	−0.37**	−0.46**
WE t1	0.32**	0.27**	0.58**	0.35**	0.39**	0.20*	0.59**	0.37**	0.10	0.13	0.17	0.01	—		WE t3-EE t3 −0.47**	
WE t2	0.12	0.33**	0.35**	0.55**	0.19	0.45**	0.37**	0.54**	0.27**	0.18	0.19	0.21*	0.55**	—		
WE t3	0.21*	0.34**	0.30**	0.42**	0.20*	0.32**	0.30**	0.42**	0.28**	0.09	0.06	0.22*	0.53**	0.65**	−0.28**	−0.32**
EE t1	−0.18	−0.07	−0.47**	−0.35**	−0.44**	−0.34**	−0.43**	−0.33**	−0.16	−0.29**	−0.59**	−0.27**	−0.40**	−0.42**	—	
EE t2	0.12	−0.14	−0.15	−0.38**	−0.24*	−0.48**	−0.11	−0.32**	−0.11	−0.22*	−0.51**	−0.62**	−0.15	−0.41**	0.52**	—
EE t3	0.02	−0.12	−0.14	−0.28**	−0.19	−0.34**	−0.11	−0.24*	−0.07	−0.24*	−0.47**	−0.52**	−0.16	−0.42**	0.52**	0.67**

ACS = applicability of character strengths, CIT = thriving, SWB = subjective well-being, PWB = psychological well-being, PCS = physical component summary, MCS = mental component summary, WE = work engagement, EE = emotional exhaustion; t1/t2/t3 = first/second/third time point (*p < 0.05, **p < 0.01; all two-tailed tests).

**FIGURE 1:**
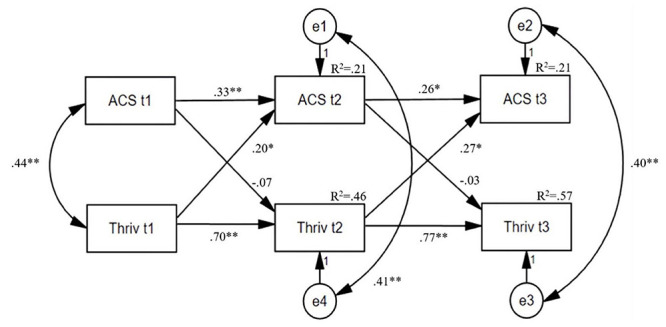
Cross-lagged-path analysis of the applicability of signature character strengths (ACS) and thriving (Thriv) across the three time points (t1, t2, t3). Standardized values *p < 0.05 and **p < 0.01; n.s., not significant.

The first hypothesis that there is a positive effect of the applicability of signature character strengths on well-being (thriving, SWB, PWB) and health (physical and mental health) over time, had to be rejected because inverted effects were found. The longitudinal cross-lagged panel analyses (Figure 1 and Table 3; exemplary) revealed reverse significant positive effects of well-being on signature strengths’ applicability at later time points. This reverse effect was significant for thriving [*β* = 0.20 (t1-t2); *β* = 0.27 (t2-t3)] and PWB [*β* = 0.23 (t1-t2); *β* = 0.27 (t2-t3)]. Taking a closer look at PWB, the subscales ‘relationship’ and ‘meaning’ at t1 had a significant effect on signature strengths’ applicability at t2 (*β* = 0.19|0.20). No such effects were found for SWB, physical or mental health at any time point. In particular, significant high auto-correlations over time were found for PWB [*β* = 0.72 (t1-t2); *β* = 0.77 (t2-t3)] indicating higher stability in the long-term compared to other constructs, e.g., SWB [*β* = 0.52 (t1-t2); *β* = 0.63 (t2-t3)] or the applicability of signature character strengths [*β* = 0.32-0.42 (t1-t2); *β* = 0.25-0.40 (t2-t3)] being potentially more shapeable by external circumstances and the environment. From t1 to t2, and t2 to t3, mental health correlated higher than physical health [*β* = 0.42 vs. 0.25 (t1-t2); *β* = 0.50 vs. 0.33 (t2-t3)]. The standardized regression weights (*β*) of all multiple linear regression analyses can be found in Table 3.

**TABLE 3:** Results from the longitudinal cross-lagged panel designs using multiple regression analyses.

**Crit.**	**Predict.**	***β***	***p***	***R*^2^**	**Crit.**	**Predict.**	***β***	***p***	***R*^2^**	**Crit.**	**Predict.**	***β***	***p***	***R*^2^**	**Crit.**	**Predict.**	***β***	***p***	***R*^2^**	**Crit.**	**Predict.**	***β***	***p***	***R*^2^**
ACS t2	ACS t1	**0.33**	0.001	0.21	ACS t2	ACS t1	**0.40**	<0.001	0.18	ACS t2	ACS t1	**0.32**	0.002	0.22	ACS t2	ACS t1	**0.42**	<0.001	0.18	ACS t2	ACS t1	**0.42**	<0.001	0.18
	CIT t1	**0.20**	0.046			SWB t1	0.07	0.480			PWB t1	**0.23**	0.026			MCS t1	0.06	0.535			PCS t1	−0.05	0.587	
CIT t2	ACS t1	−0.07	0.437	0.46	SWB t2	ACS t1	−0.07	0.421	0.26	PWB t2	ACS t1	−0.04	0.617	0.49	MCS t2	ACS t1	−0.16	0.086	0.19	PCS t2	ACS t1	0.09	0.384	0.07
	CIT t1	**0.70**	<0.001			SWB t1	**0.52**	<0.001			PWB t1	**0.72**	<0.001			MCS t1	**0.42**	<0.001			PCS t1	**0.25**	0.012	
ASC t3	ASC t2	**0.26**	0.012	0.21	ASC t3	ASC t2	**0.33**	<0.001	0.18	ASC t3	ASC t2	**0.25**	0.016	0.21	ASC t3	ASC t2	**0.39**	<0.001	0.15	ASC t3	ASC t2	**0.40**	<0.001	0.15
	CIT t2	**0.27**	0.010			SWB t2	0.18	0.067			PWB t2	**0.27**	0.010			MCS t2	0.02	0.876			PCS t2	−0.05	0.625	
CIT t3	ASC t2	−0.03	0.693	0.57	SWB t3	ASC t2	−0.02	0.853	0.38	PWB t3	ASC t2	−0.03	0.751	0.57	MCS t3	ASC t2	0.01	0.879	0.25	PCS t3	ASC t2	−0.11	0.276	0.12
	CIT t2	**0.77**	<0.001			SWB t2	**0.63**	<0.001			PWB t2	**0.77**	<0.001			MCS t2	0.50	<0.001			PCS t2	**0.33**	<0.001	

*Crit. = criteria, Predict. = predictor, *β* = standardized beta-regression weight, *p* = level of significance, *R^2^* = coefficient of determination, ACS = applicability of character strengths, CIT = thriving, SWB = subjective well-being, PWB = psychological well-being, MCS = mental component summary, PCS = physical component summary; t1/t2/t3 = first/second/third time point. Bold = significant regression weights (ß).*

The second hypothesis on indirect effects via work engagement and emotional exhaustion was partly confirmed. The mediation analyses revealed some indirect effects via work engagement (*a^∗^b* = 0.06 to 0.16; all CI’s excluding number ‘zero’) but none via emotional exhaustion (Table 4). At all three time points, indirect effects via work engagement emerged between the relation of the applicability of signature character strengths and thriving, SWB, and PWB, whereas indirect effects via work engagement on mental and physical health were only evident at t2 (both) and t3 (mental health; Table 4).

**TABLE 4:** Mediation analyses of work engagement and emotional exhaustion on the relation of applicability of signature character strengths and thriving, subjective and psychological well-being, mental and physical health at the three time points.

**Paths**		***a***	***b***	***c***	***c‘***	a*b	**95% CI**
ACS→WE→CIT	t1 t2 t3	0.32** 0.33** 0.44**	0.48** 0.43** 0.34**	0.43** 0.48** 0.53**	0.28** 0.34** 0.38**	0.15* 0.15* 0.15*	[ 0.067, 0.30 ] [ 0.057, 0.27 ] [ 0.058, 0.27 ]
ACS→WE→SWB	t1 t2 t3	0.32** 0.33** 0.44**	0.30** 0.37** 0.22*	0.26** 0.30** 0.35**	0.16 0.18* 0.25*	0.10* 0.12* 0.10*	[ 0.024, 0.23 ] [ 0.038, 0.25 ] [ 0.009, 0.21 ]
ACS→WE→PWB	t1 t2 t3	0.32** 0.33** 0.44**	0.50** 0.43** 0.35**	0.46** 0.51** 0.54**	0.30** 0.37** 0.38**	0.16* 0.14* 0.16*	[ 0.065, 0.31 ] [ 0.057, 0.26 ] [ 0.061, 0.27 ]
ACS→WE→MCS	t1 t2 t3	0.32** 0.33** 0.44**	0.16 0.17 0.25*	0.11 0.13 0.16	0.05 0.01 0.05	0.05 0.06* 0.11*	[ −0.012, 0.16 ] [ 0.002, 0.16 ] [ 0.022, 0.23 ]
ACS→WE→PCS	t1 t2 t3	0.32** 0.33** 0.44**	0.09 0.19 0.11	0.06 0.06 0.07	0.03 0.01 0.02	0.03 0.06* 0.05	[ −0.019, 0.10 ] [ 0.010, 0.16 ] [ −0.032, 0.14 ]
ACS→EE→CIT	t1 t2 t3	−0.18 −0.14 −0.17	−0.39** −0.23** −0.28**	0.43** 0.06 0.53**	0.36** 0.02 0.48**	0.07 0.03 0.05	[ 0.000, 0.19 ] [ −0.009, 0.11 ] [ −0.005, 0.14 ]
ACS→EE→SWB	t1 t2 t3	−0.18 −0.14 −0.17	−0.37** −0.41** −0.38**	0.26** 0.30** 0.35**	0.19* 0.24** 0.28**	0.07 0.09 0.06	[ −0.003, 0.20 ] [ −0.013, 0.15 ] [ −0.009, 0.17 ]
ACS→EE→PWB	t1 t2 t3	−0.18 −0.14 −0.17	−0.37** −0.25** −0.22*	0.46** 0.51** 0.54**	0.39** 0.48** 0.50**	0.06 0.04 0.04	[ 0.000, 0.18 ] [ −0.006, 0.10 ] [ −0.002, 0.13 ]
ACS→EE→MCS	t1 t2 t3	−0.18 −0.14 −0.17	−0.62** −0.57** −0.55**	0.11 0.13 0.16	−0.01 0.05 0.07	0.11 0.08 0.09	[ −0.015, 0.27 ] [ −0.210, 0.19 ] [ −0.015, 0.21 ]
ACS→EE→PCS	t1 t2 t3	−0.18 −0.14 −0.17	−0.15 −0.23* −0.09	0.06 0.06 0.07	0.03 0.02 0.06	0.03 0.03 0.01	[ −0.009, 0.13 ] [ −0.003, 0.12 ] [ −0.007, 0.08 ]

All standardized values. a = direct effect of ACS on the mediator variable; b = direct effect of the mediator variable on outcome; c = total effect of ACS on outcome; c‘ = direct effect of ACS on outcome; a*b = indirect effect; CI = confidence interval; ACS = applicability of character strengths, WE = work engagement; CIT = thriving, SWB = subjective well-being, PWB = psychological well-being, MCS = mental component summary, PCS = physical component summary, EE = emotional exhaustion; t1/t2/t3 = first/second/third time point *p < 0.05 and **p < 0.01.

**FIGURE 2:**
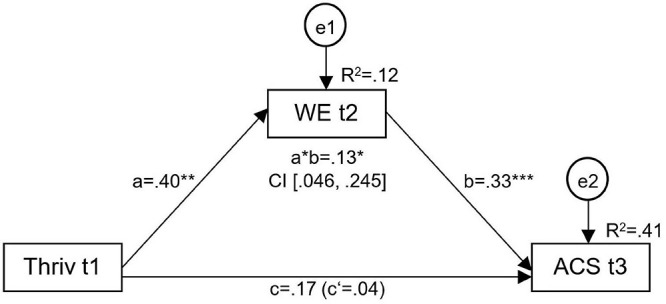
Mediation analysis over time revealing a significant indirect effect via work engagement (WE t2) on the relation of thriving (Thriv t1) and the applicability of signature character strengths (ACS t3), controlled for ACS t1. a = direct effect of CIT t1 on the mediator variable; b = direct effect of the mediator variable on ACS t3; c = total effect of CIT t1 on ACS t3; c’ = of CIT t1 on ACS t3; a*b = indirect effect of CIT t1 and ACS t3; R^2^ = coefficient of determination; *p < 0.05, **p < 0.01, and ***p < 0.001.

Due to the detected reverse significant positive effects of well-being on signature strengths’ applicability at later time points and the mediation effects via work engagement, an additional mediation analysis with thriving (t1), work engagement (t2) and the applicability of signature character strengths (t3) over time was tested (Figure 2). The results showed significant direct effects of thriving on work engagement (*a* = 0.40), of work engagement on the applicability of signature character strengths (*b* = 0.33) and a significant indirect effect (*a^∗^b* = 0.13; CI [0.046, 0.245]), even when controlling for the applicability of signature character strengths at t1. There was no significant direct or total effect of thriving (t1) on the applicability of signature character strengths (t3) further underlining the importance of work engagement as mediator over time. Considering this fact, thriving can be interpreted as a long-term predictor for the applicability of signature character strengths over 2 years. According to the original hypothesis that the applicability of signature character strengths (t1) affects thriving (t3), no effect was found in this long-term mediation analysis (*c* = 0.10, *p* = 0.32; *c’* = 0.11, *p* = 0.22). However, one significant direct effect was found for work engagement (t2) on thriving (t3; *b* = 0.33, *p* < 0.001).

## Discussion

Future health care professionals like medical students belong to an at-risk group for impaired well-being due to very high study demands. This study examined potential protective effects of the applicability of signature character strengths over time. The longitudinal cross-lagged panel analyses revealed unexpected significant positive effects of thriving and PWB on signature strengths’ applicability at later time points indicating that higher levels of well-being might be mandatory first to perceive applicability at all and to have access to one’s own signature strengths. This significant result of a reverse longitudinal relation attracts attention and inspires one to think differently. So far, strengths-based intervention studies (e.g., Seligman et al., 2005; Gander et al., 2013) found effects the other way round. By conducting the intervention ‘Use your strengths every day in a new way’ participants were able to reduce depressive symptoms within 6 months (Seligman et al., 2005), whereas happiness increased by almost all positive interventions compared to baseline in a time span of 6 months (Gander et al., 2013). Cross-sectionally, significant relations of signature character strengths’ applicability with well-being and mental health (Hausler et al., 2017a) or in particular with PWB and work engagement (Huber et al., 2019) were shown. In the present study, no comparable effects were found and according to these results, there could be a long-term effect the other way round as previously postulated.

Current research focused preliminary on investigating influences of (signature) character strengths on well-being, health, or other dependent variables. A reverse hypothesis-being consistent with findings from this study-that higher levels of well-being could lead to more perceived applicability of signature character strengths has never been formulated and tested before. The ‘broaden-and-build’ theory (Fredrickson, 2011) might be helpful for interpreting these novel results. This theory states that certain discrete positive emotions (joy, interest, contentment, pride, and love) share the ability to broaden one’s momentary thought-action repertoires in terms of consciousness, latitude, and thinking. In the long run, this extension will result in building new enduring resources and developing skills and abilities in a new way. Therefore, positive feelings can lead to a more open-minded and positive basic adjustment and consequently, to mental, social, and physical strength. Evidence for this assumption arises from two decades of experiments conducted by Isen (2000). They have shown that positive affect is associated with mind patterns that are notably unusual, flexible, creative, integrative, open to information, and efficient. Moreover, the researchers found an increased preference for variety and acceptance of a broader array of behavioral options. As such effects were not found longitudinally for SWB within this sample, one could argue that positive experiences by other more stable components of PWB, like support, community, engagement, self-efficacy or meaning, might have broaden the students’ minds and promoted their perceived applicability of signature character strengths over time. As no significant changes over time concerning the mean levels of thriving, SWB or PWB were found, possible gain spirals between well-being and the applicability of signature character strengths can rather be excluded. Conversely, but in terms of the broadening hypothesis, negative states (e.g., anxiety, depression, failure) would narrow people’s minds by inducing specific action tendencies characterized by defensive and/or evolutionary survival values. As medical students often report distress (e.g., Kötter et al., 2017), depressive symptoms, and emotional exhaustion (Dyrbye et al., 2008; 2014), directed strategies regulating negative emotions by causing positive ones could be beneficial as they might correct or undo the after effects of negative emotions (‘undoing hypothesis’; Fredrickson, 2011). Based on the results of this study and using the ‘broaden and built’ theory to explain these novel findings, future research should directly address whether and how interventions aiming to increase subjective or psychological well-being lead to an increased (implicit) uptake of one’s signature character strengths. In particular, focusing on medical students with low levels of thriving might benefit from an approach focusing on well-being first, as they perceived it harder to access and apply their own signature character strengths.

Another approach to interpret these novel results has been already discussed by Feist et al. (1995), who investigated the so-called ‘bottom-up’ and ‘top-down’ theories of SWB. Bottom-up theories claim that by merely collecting positive experiences in particular domains (e.g., family, marriage, work, education, etc.) people develop an overall sense of well-being being defined as ‘state-like’ (Diener and Ryan, 2009). In contrast, the top-down view assumes that people have a predisposition to interpret life experiences in either positive or negative ways, which in turn colors one’s evaluation of satisfaction in various domains. In this theory, well-being was defined ‘trait-like’ describing a more positive reaction to the environment in general where individual experiences are not objectively good or bad but their interpretations are. Feist et al. (1995) tested possible direct and indirect effects of physical health, daily hassles, constructive thinking, and world assumptions on SWB (bottom up) and vice versa (top down). Results showed that both models provided good fit with neither model providing a closer fit than the other, suggesting that SWB can operate either bottom-up or top-down. Other top-down models on physical health have been included in well-being-related domains (optimism: Scheier and Carver, 1985; negative affectivity: Watson and Clark, 1984) showing that general well-being dispositions can filter the perception of daily experiences. This observational study revealed in particular long-term effects of PWB on the applicability of signature character strengths. PWB showed stable and consistently strong correlations across all three time points, indicating mechanisms toward a top-down rather than a bottom-up theory. This further supports and potentially extends the ‘broaden-and-build’ theory in terms of perceiving more positive emotions (‘trait-like’) to use one’s own resources or to build new enduring resources.

Alternatively, the results can also be explained by the so-called ‘set point-theory’ (e.g., Headey, 2008). This theory was originally developed to explain why repeated dieting is unsuccessful in producing long-term change in body weight or shape. This idea was then transferred to the field of psychology and SWB, stating that individuals have differing but mostly stable levels of SWB (substantially evolving from personality traits and other factors-hereditary or determined in early life) and that major life events can cause deviations from this well-being ‘set point.’ Their effects are usually transitory and, after a period of ‘deviation’, people return to their previous set points regardless of the direction of deviation. Based on the comparatively high levels (‘set points’) of SWB and PWB in this sample, the perceived applicability of signature character strengths might have been not powerful enough to statistically influence or move the respective well-being set points in a naturalistic setting. This could also explain why participants of strength-based intervention studies, which intentionally focus on applying character strengths, enhance well-being at later time points (e.g., Seligman et al., 2005; Gander et al., 2013; Proyer et al., 2014).

In the present study, work engagement turned out to be a significant mediator at each time point for the association between the applicability of signature character strengths and thriving as well as SWB and PWB separately. Indirect effects on mental and physical health were not consistent across the three time points. The positive effect of having the meaningful opportunity or even demand to potentially or actually apply one’s own signature character strengths at work (= applicability) on work engagement has been already shown by Strecker et al. (2019). There, the applicability of signature character strengths mediated the effect of ‘thriving’ work characteristics on work engagement of hospital physicians. Results showed that some work characteristics (like autonomy) predicted the applicability of signature character strengths at work, but also the applicability in turn was able to predict work characteristics (like social support by colleagues and supervisors). According to the JD-R model, which distinguishes between job demands associated with physical and/or mental ‘costs’ (e.g., adverse physical environment, stressful emotional interactions, time pressure) and ‘resources’ (e.g., autonomy, feedback, social support), motivation (work engagement) can be increased by resources such as applicable character strengths (Bakker and Demerouti, 2007). The long-term mediation analysis corresponding to the reverse effects of thriving and PWB on the applicability of signature character strengths further underlined the importance of work engagement being a mediator over time. It seems that this type of motivation in medical studies is necessary in the long run to build new enduring resources and develop skills and abilities in a new way, like in a first step perceiving signature character strengths’ applicability. The inconsistent indirect effects via work engagement on mental health could be a result of study-specific conditions. Most of the medical students at the first time point were at the end of their first semester, at the second time point they were at the beginning or in the middle of their first clinical electives (third/fourth semester), and at the third time point they had already finished their clinical electives (fifth/six semester). These three periods characterize potentially different phases of adjustment to changing demands of the medical study: the phase of extensive learning before an important exam, and the phase of starting and finishing practical work depending on their medical knowledge and skills. Consequential, respective different levels of demands and engagement arise that might have (indirectly) influenced medical students’ health. If study demands constantly increase and possibilities to apply individual strengths decrease or disappear, the risk of burnout is increased as well (Hakanen et al., 2008).

The hypothesized indirect effect via reduced emotional exhaustion was not found. There were no mediating effects at any time point. A longitudinal 3-year study with dentists also based on the JD-R model, revealed quite small effects of job resources (like the applicability of signature character strengths) on burnout whereas job demands significantly predicted burnout and on the long-run depression (Hakanen et al., 2008). In contrast, a cross-sectional study with medical students (e.g., Hausler et al., 2017a) revealed indirect effects via the burnout dimension emotional exhaustion. Means of the variable ‘emotional exhaustion’ in Hausler’s study (*N* = 387) did not significantly differ from this study at the first time point. Therefore, the reduced sample size in the second and third wave of data collection might be a crucial factor for the ‘missing’ indirect effects found in the cross-sectional study.

### Limitations

One potential limitation of this study is the sample size in the longitudinal calculations. However, post-hoc G^∗^power analyses revealed a sufficient sample size to identify at least medium effects with an adequate statistical power > 80% and *p* < 0.05 (common for social sciences; Faul et al., 2009). Concerning the dropout rate, the very comprehensive online test battery could be one explanation as many students did not finish all questionnaires or did not participate again at the second and/or third time point. Furthermore, medical students only participating at t1 reported significantly lower physical health than students taking part in the study several times. However, the mean values of the PCS scale were within the normal range and therefore not clinically relevant. 2 years is a quite long period to track the same students and often problems arise in terms of keeping them at it. The workload and study demands vary extensively across this period and potential influences on the applicability of signature character strengths are difficult to observe. The offered incentives (individual feedback on their signature character strengths, medical education credits, raffle of medical books and vouchers) were not triggering as much motivation as desired. Generally, participants are more likely to exercise their free will in online studies than in in-lab studies, resulting in higher dropout rates (Dandurand et al., 2008). When it comes to longitudinal study designs, the decreasing number of participants due to dropouts is a typical problem. However, 19% completion rate over a period of 2 years (meaning almost 300 assessments) is satisfactory in terms of post-hoc power analyses (see above). The degree to which these results can be generalized to other (medical) students is limited and other universities in different cultural settings should be investigated to verify these results for educational settings in different countries.

Another limitation may result from the used measures. For example, the SF-12 only gives general information on physical and mental health but not on specific scales and maybe not all items are suitable for younger people (e.g., limited health in moderate activities such as moving a table or playing golf, or climbing several flights of stairs). Due to the different weighted regression coefficients always included in the standardized sum scores, it was not possible to calculate internal consistencies for the respective sample, making a well-grounded evaluation even more difficult. Even though most Cronbach’s alphas of the VIA-120 measuring individual character strengths were acceptable, some scales were not consistent across various time points. Comparatively low internal consistencies were also found for the ACS-RS, particularly at t3. This comparatively low reliability of the ACS-RS-measure may have led to an under-estimation of effects and reduced statistical power impeding the possibility to identify only small, but significant effects (e.g., Hopkins and Hopkins, 1979). Maybe this has contributed to the fact that the hypothesized lagged effects of the applicability of signature character strengths on well-being were not identified in this data set. However, the comparative low reliability of the ACS-RS may also have led to an underestimation of the found significant lagged (reversed) effects of well-being on the applicability of signature character strengths, which seems to be the more innovative result of the present study. In general, all data analyzed are student self-reports possibly including bias due to single sources (e.g., mood), item characteristics / context (e.g., length of scales) or the data collection context (e.g., identical tools presented online) being subsumed under the term ‘common method bias’, which could have influenced the results. Furthermore, type I errors might have occurred due to partial multicollinearity of the study variables and a comparatively small sample size both increasing the possibility of capitalization on chance.

Finally, a clear separation of work (= study) and private life is very difficult as students tend to keep themselves busy with their studies even in private life and boundaries between life domains are frequently blurred. Therefore, a more precise statement beyond the construct of applicability would be helpful with a more in depth separation of both contexts.

### Implications

Results demonstrated significant longitudinal effects of thriving in general and PWB in particular on the applicability of signature character strengths. The mediating role of work engagement has been highlighted pointing to the importance of signature character strengths at work. The medical study and the work as physician later on imply constantly great challenges. High mental strain resulting in higher risk for burnout are part of medical students’ (future) jobs. Therefore, this endangered group could benefit from a stronger focus on improving their well-being and applying their signature character strengths already during their studies. Beside the signature character strengths perceiving the highest applicability in this sample (*fairness, hope, kindness, perseverance, zest*) and possible corresponding specific interventions, students should be informed about the positive effects of strengths in general as well as about their role in terms of thriving and work engagement. In a further step, the identification and appropriate application of one self’s signature character strengths could be already implemented into the medical curriculum in accordance with the JD-R model. For example, teaching medical students that paying attention to their well-being is important as it can lead to a higher applicability of signature character strengths later on. Applying one’s signature character strengths in turn can increase personal resources, boosting well-being and health for their future work life. Medical universities should be prepared to facilitate their students’ well-being, work engagement and pursue to replicate possibilities for signature character strengths application, striving for better studying conditions in terms of the third pillar of Positive Psychology (positive institutions). Consistent with the ‘broaden-and-build’ theory, medical students will benefit from such contents during education to become more open-minded and to develop a more positive basic adjustment already in younger years. These positive effects could possibly result in increased work engagement and decreased burnout risk later on.

## Conclusion

This observational study reveals significant longitudinal positive effects of thriving in general and PWB in particular of medical students on their signature character strengths’ applicability at later time points with consistently indirect effects via work engagement. A certain level of individual well-being may be a necessary prerequisite to being able to access and apply one’s character strengths outside an interventional or therapeutic setting. Therefore, ensuring well-being of medical students throughout their studies should be on the agenda of every curriculum developmental plan. Subsequent longitudinal studies should replicate the detected novel relations between all constructs in different samples and particular different cultures. Different influences of study demands and conditions, arguing for or against possible gain spirals of well-being and the applicability of signature character strengths should be explored.

## Data Availability Statement

The datasets generated for this study are available on request from the corresponding author.

## Ethics Statement

The studies involving human participants were reviewed and approved by The Board for Ethical Questions in Science of the University of Innsbruck. The participants provided their written informed consent to participate in this study.

## Author Contributions

AH, CS, TH, and SH were substantially involved in planning and conducting the study. AB provided a basis for this manuscript and carried out the data analyses. AH drafted the article. All authors revised the manuscript critically for important intellectual content, read and approved the submitted version.

## Conflict of Interest

The authors declare that the research was conducted in the absence of any commercial or financial relationships that could be construed as a potential conflict of interest.
